# Adjunctive Thymosin Beta-4 Treatment Influences MΦ Effector Cell Function to Improve Disease Outcome in *Pseudomonas aeruginosa*-Induced Keratitis

**DOI:** 10.3390/ijms222011016

**Published:** 2021-10-13

**Authors:** Yuxin Wang, Thomas W. Carion, Abdul Shukkur Ebrahim, Gabriel Sosne, Elizabeth A. Berger

**Affiliations:** Department of Ophthalmology, Visual & Anatomical Sciences, Wayne State University School of Medicine, 540 E. Canfield Avenue, Detroit, MI 48201, USA; gp8667@wayne.edu (Y.W.); tcarion@med.wayne.edu (T.W.C.); eabdulsh@med.wayne.edu (A.S.E.); gsosne@med.wayne.edu (G.S.)

**Keywords:** Tβ4, MΦ, immunoregulation, keratitis, RNS, efferocytosis

## Abstract

Our previous work has shown that topical thymosin beta 4 (Tβ4) as an adjunct to ciprofloxacin treatment reduces inflammatory mediators and inflammatory cell infiltrates (neutrophils/PMN and macrophages/MΦ) while enhancing bacterial killing and wound healing pathway activation in an experimental model of *P. aeruginosa*-induced keratitis. This study aimed to mechanistically examine how Tβ4 influences MΦ function in particular, leading to reduced inflammation and enhanced host defense following *P. aeruginosa*-induced infection of the cornea. Flow cytometry was conducted to profile the phenotype of infiltrating MΦ after infection, while generation of reactive nitrogen species and markers of efferocytosis were detected to assess functional activity. In vitro studies were performed utilizing RAW 264.7 cells to verify and extend the in vivo findings. Tβ4 treatment decreases MΦ infiltration and regulates the activation state in response to infected corneas. MΦ functional data demonstrated that the adjunctive Tβ4 treatment group significantly downregulated reactive nitrogen species (RNS) production and efferocytotic activity. In addition, the in vitro studies showed that both Tβ4 alone and adjunctive Tβ4 treatment influenced MΦ cellular function following LPS stimulation. Collectively, these data provide further evidence that adjunctive Tβ4 + ciprofloxacin treatment offers a more efficacious option for treating bacterial keratitis. Not only does the adjunctive therapy address both the infectious pathogen and corneal wound healing response, but it also influences MΦ infiltration, activation, and function, as revealed by the current study.

## 1. Introduction

Bacterial keratitis is a rapidly progressing, sight-threatening infection of the cornea that can lead to corneal opacification, perforation, and endophthalmitis. *Pseudomonas aeruginosa* is one of the most common pathogens identified in bacterial keratitis with an increasing frequency, especially among contact lens wearers and immunocompromised individuals [1,2,3]. Keratitis-induced corneal opacification is among the leading causes of blindness worldwide and results in a substantial economic burden on families, hospitals, and societies [4,5]. Current antibiotic treatment for bacterial keratitis mainly addresses the pathogen with fourth-generation ophthalmic fluoroquinolone as the preferred choice for treating *P. aeruginosa*-infected corneas [6,7]. Although controlling bacteria in keratitis is very important, clinical outcomes can still be inferior due to host-induced corneal opacification that remains unresolved. Restoration of immune homeostasis following infection is an integral part of ocular function [8]. Corticosteroid treatment addresses the host response to some extent; however, its use is not substantiated and usually is not recommended [9,10]. Therapies are needed to both regulate the inflammatory response and promote corneal wound healing to properly resolve the visual disturbance. 

Tβ4 is a small, naturally occurring 43-amino acid protein that is highly conserved across species and is endogenously expressed in all tissues and cell types, except red blood cells, with a high concentration detected within platelets and wound fluid [11,12]. Initially, Tβ4 was thought to act solely as an actin sequestering molecule, but now is commonly recognized for its importance in inflammation and wound healing. In the cornea, Tβ4 has been found to promote epithelial cell migration, inhibit epithelial cell apoptosis, decrease PMN infiltration after chemical injury, modulate inflammatory cytokines/chemokines, and downregulate TNF-α-stimulated NF-κB activation in corneal epithelial cells [12,13,14]. Safety profiles from clinical trials for the treatment of dry eye disease and neurotrophic keratopathy have not indicated any adverse effects with Tβ4 treatment [15,16,17,18]. 

The inflammatory process is usually well-regulated by the signals that initiate and maintain inflammation and signals that shut down or resolve the process. An imbalance between the two signals leaves inflammation poorly modulated, resulting in chronicity and tissue destruction [19]. MΦ are a major component of the mononuclear phagocyte system derived from the bone marrow, including blood monocytes and tissue MΦ. When infection occurs, both tissue-resident MΦ and recruited monocytes undergo activation and incite inflammation to fight against the pathogen [20]. MΦ have three principal functions in inflammation: antigen presentation, phagocytosis/efferocytosis, and immunomodulation through the expression of different cytokines and regulatory factors [21]. Removing or deactivating inflammatory mediators and effector cells in a timely manner suppresses inflammation and therefore enables the host to repair damaged tissues and restore homeostasis. MΦ produce a wide range of biologically active molecules that contribute to the autoregulatory loop of the inflammatory process [22] and play critical roles from the induction of inflammation to the initiation of resolution. The ideal outcome of inflammation is complete resolution [19], where apoptosis of PMN and subsequent clearance of apoptotic cells and cellular debris by MΦ are critical to resolving inflammation [23]. However, the problem in microbial keratitis is that the infiltration of inflammatory cells (e.g., PMN and MΦ) becomes overwhelming, and the host response shifts toward uncontrolled [24,25]. This dysregulated immune response is usually accompanied by the generation of large amounts of reactive species [26]. Studies have illustrated that Tβ4 has anti-inflammatory and immunoregulatory functions, which help improve the outcomes of ocular inflammation [14,27]. Our previous work has demonstrated the importance of Tβ4 as a therapeutic agent in conjunction with ciprofloxacin in *P. aeruginosa*-induced keratitis [28]. Little published literature exists regarding the mechanism(s) of Tβ4 function on inflammatory cells after bacterial infection. This study is an extension of our previous work and investigates the mechanisms by which adjunctive Tβ4 treatment impacts MΦ function using the well-established *Pseudomonas* keratitis model. 

## 2. Results

### 2.1. Disease Response following P. aeruginosa-Induced Infection in B6 Mice

**Figure 1:**
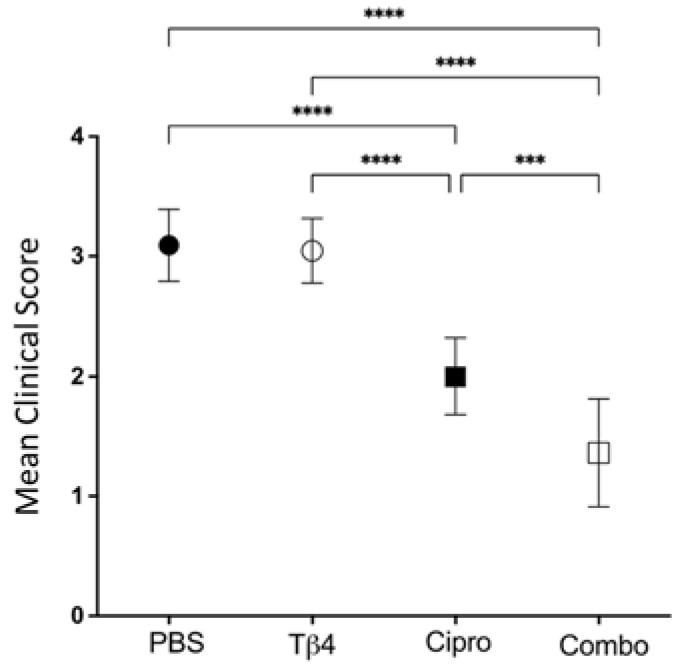
Disease response presented as mean clinical score. Ocular disease response of *P. aeruginosa*-infected B6 mice was graded at 3 days p.i. and results are represented as mean clinical scores ± SD. Treatment groups included: PBS control (●), Tβ4 (○), ciprofloxacin (■), and Tβ4 + ciprofloxacin as a combo (□). (*n* = 11 corneas/group). *** *p* < 0.001; **** *p* < 0.0001.

Firstly, clinical scores were recorded to assess the disease response at 3 days post-infection (p.i.), as shown in Figure 1. Mean clinical scores were similar between Tβ4-treated mice and PBS controls. Disease response was significantly improved in ciprofloxacin-treated corneas when compared to both PBS controls and Tβ4-treated mice. However, the Tβ4 + ciprofloxacin-treated animals exhibited the lowest clinical scores and the most improved disease outcome, with excellent resolution of corneal opacity at 3 days p.i.

### 2.2. Flow Cytometric Analyses of MΦ Infiltrates in P. aeruginosa-Infected B6 Mice

**Figure 2:**
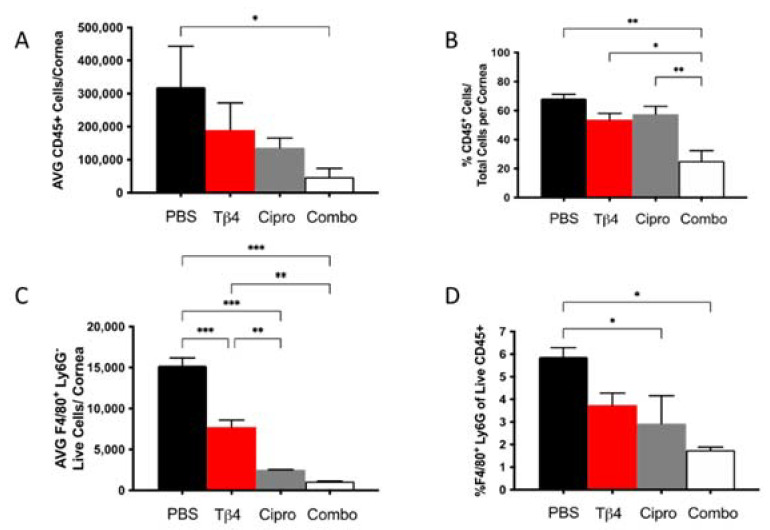
Flow cytometry results of inflammatory cellular infiltrates following infection. CD45^+^ leukocytes (**A**,**B**) and CD45^+^F4/80^+^Ly6G^−^ MΦ (**C**,**D**) as determined by flow cytometry at 3 days p.i. in corneas of PBS-, Tβ4-, cipro-, and combo-treated B6 mice. Results are shown as averages of single cell populations (**A**,**C**), % of leukocytes per total cells per cornea (**B**), or % of live MΦ per total population of live leukocytes (**D**). *n* = 5 corneas/group; * *p* < 0.05, ** *p* < 0.01, *** *p* < 0.001.

Our previous work has suggested that adjunctive Tβ4 treatment influences MΦ function in *P. aeruginosa*-infected corneas [28]. We then assessed the MΦ population in infected corneas for each treatment group at 3 days p.i. using flow cytometry. First, increasingly fewer CD45^+^ leukocytic cells were present in infected corneas of Tβ4-, ciprofloxacin-, and combination-treated mice when compared to PBS controls (Figure 2A), though only the adjunctive Tβ4 treatment group was significantly decreased compared to the PBS control. When analyzed as % of CD45^+^ cells/total corneal cells (Figure 2B), there were no statistical differences between PBS control, Tβ4, and ciprofloxacin groups. Corresponding with absolute numbers, the % of CD45^+^ cells was significantly decreased in the adjunctive Tβ4 treatment group compared to all other groups. 

From the CD45^+^ gated population, inflammatory cell infiltrates were further characterized to provide live MΦ (CD45^+^/F480^+^/Ly6G^−^) single-cell populations, as shown in Figure 2 (panels C,D). Corneas of Tβ4, ciprofloxacin, and adjunctive Tβ4 treatment groups had significantly fewer MΦ when compared to the PBS control group (Figure 2C). Moreover, absolute numbers of live MΦ in the ciprofloxacin and combination treatment groups were further decreased (by approximately 3–4-fold) when compared to Tβ4 alone. No difference was detected in absolute MΦ numbers between adjunctive Tβ4 and ciprofloxacin groups. The % of MΦ/total CD45^+^ cells revealed that all three treatment groups trended downward, but only ciprofloxacin and adjunctive Tβ4 treatment groups displayed statistical differences when compared to the PBS control (Figure 2D). By assessing leukocytic infiltration in these four different treatment groups, adjunctive Tβ4 therapy effectively reduced both CD45^+^ cells and MΦ infiltration into corneas of *P. aeruginosa*-infected B6 mice. 

### 2.3. Adjunctive Tβ4 Treatment Influences Phenotypic Profiles of MΦ Infiltrates in the Infected Cornea

**Figure 3:**
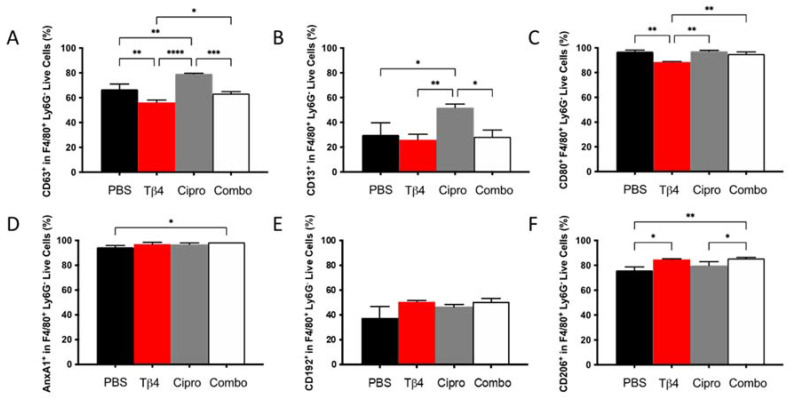
Cell surface marker expression as detected on MΦ by flow cytometry. Expression of select pro-inflammatory molecules CD63 (**A**), CD13 (**B**), CD80 (**C**) and anti-inflammatory molecules AnxA1 (**D**), CD192 (**E**), CD206 (**F**) was assessed from MΦ subpopulations in corneas of PBS-, Tβ4-, cipro-, and combo-treated B6 mice at 3 days after infection. Results are shown as averages of singlet populations for CD45^+^F4/80^+^Ly6G^−^ MΦ. *n* = 5 corneas/group; * *p* < 0.05, ** *p* < 0.01, *** *p* < 0.001, **** *p* < 0.0001.

To next examine whether there were any phenotypic differences in the MΦ populations detected in each treatment group, select cell surface molecules that are considered either pro-inflammatory or anti-inflammatory in nature were detected using flow cytometry. As presented in Figure 3, the % of cells expressing the three pro-inflammatory cell surface markers, CD63 (A), CD13 (B), and CD80 (C) within the MΦ subpopulation, were similar between adjunctive Tβ4-treated groups and PBS controls. CD63 and CD80, however, were significantly reduced in Tβ4 only compared to PBS controls. Surprisingly, ciprofloxacin alone significantly enhanced the expression of CD63 and CD13, whereas CD80 remained unchanged. Regarding the three anti-inflammatory surface markers, annexin A1 (AnxA1) (D), CD192 (E), and CD206 (F), significant increases were observed for AnxA1 and CD206 in the combination group only and CD206 in Tβ4 alone. Furthermore, the increased expression of CD206 in the combination group was significant compared to both PBS control and ciprofloxacin groups (*p* < 0.05). No differences were detected between ciprofloxacin and PBS. These results together reflect that Tβ4 influences the activation state of MΦ in the corneas of *P. aeruginosa*-infected B6 mice, where decreased pro-inflammatory markers paired with increased anti-inflammatory markers within the MΦ subpopulation in the adjunctive treatment group enhanced the resolution of the host inflammatory response.

### 2.4. Adjunctive Tβ4 Treatment Modulates the Inflammatory Response by Inhibiting RNS Generation 

**Figure 4:**
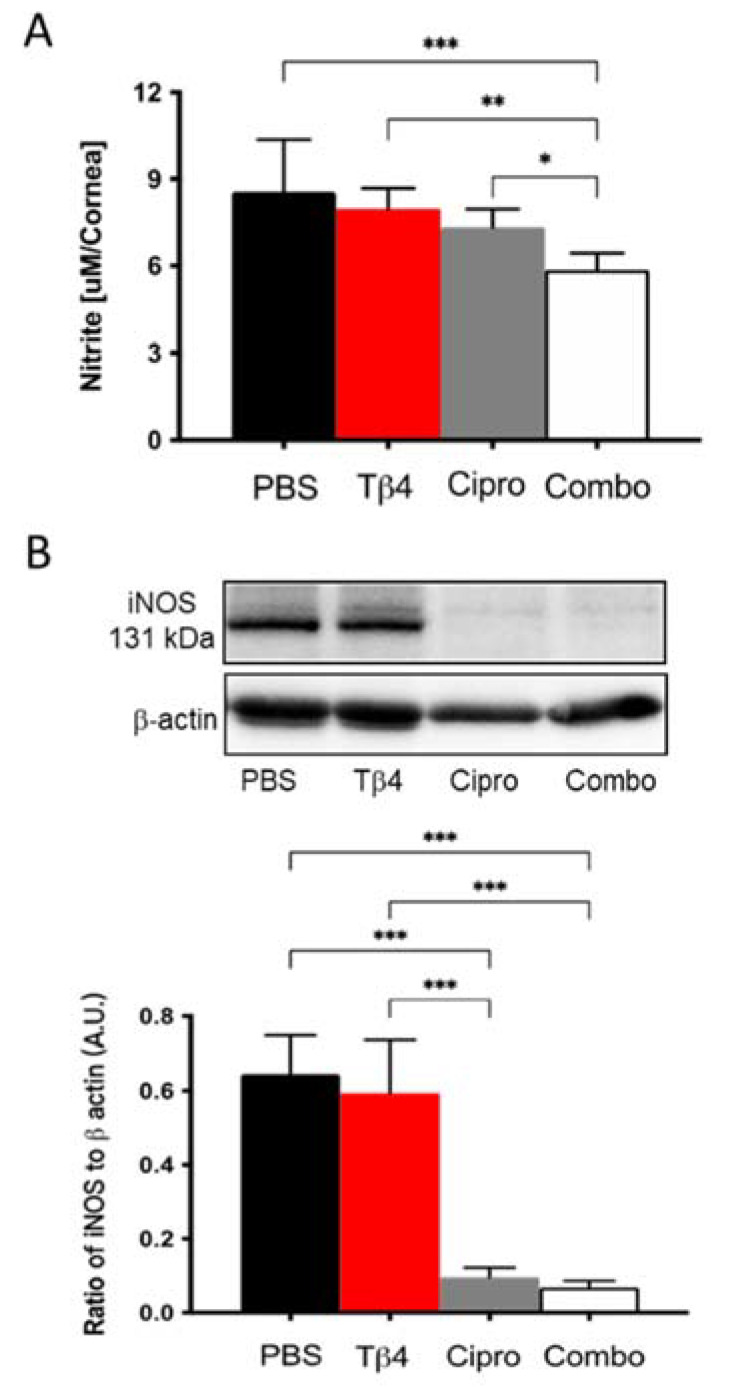
In vivo assessment of RNS generation following *P. aeruginosa*-induced corneal infection. (**A**) Nitrite concentrations were measured from corneal lysates at 3 days p.i. by the Griess assay. Results are reported as the mean concentration of nitrite (μM/cornea) ± SD. (**B**) Protein levels of iNOS were confirmed by Western blot in infected corneas at 3 days. Data shown are normalized to β-actin ± SD. *n* = 4 corneas/group; * *p* < 0.05, ** *p* < 0.01, *** *p* < 0.001.

NO levels were measured to investigate the mechanism(s) by which Tβ4 may influence MΦ cellular function. It is well-known that MΦ produce free radicals, mainly NO, one of the key mediators in response to inflammation [29]. As an indicator of MΦ activation, nitrite (a stable oxidized product of NO) was measured at 3 days p.i. (Figure 4A). Corneas from adjunctive Tβ4-treated mice exhibited significantly decreased nitrite levels compared to PBS control, Tβ4 alone, and ciprofloxacin only treatment groups. NO is produced when arginine is converted into citrulline by nitric oxide synthases (NOS). iNOS is primarily found in MΦ and is expressed maximally after an inflammatory stimulus [29]. Based on this, iNOS levels were detected at the protein level by Western blot (Figure 4B). The results indicate that both ciprofloxacin and adjunctive Tβ4 treatment significantly inhibit iNOS levels when compared to PBS control and Tβ4 only treatment. iNOS was similar between Tβ4 alone and PBS control groups. Combined with the results obtained by flow cytometry, these data further illustrate that in addition to decreasing infiltrated MΦ infiltration, cell numbers, and activation state, MΦ cellular function was inhibited by the adjunctive Tβ4 treatment.

### 2.5. Tβ4 Inhibits NO and iNOS Generation In Vitro

**Figure 5:**
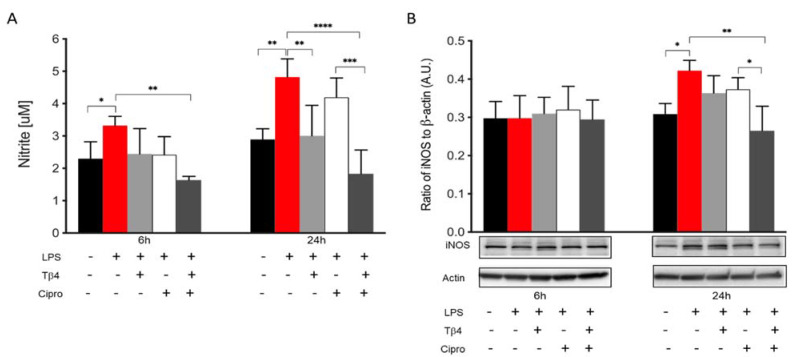
In vitro assessment of RNS generation following LPS-induced stimulation of MΦ. Nitrite (**A**) and iNOS (**B**) levels were measured from RAW 264.7 cells after 6 and 24 h of LPS stimulation. Nitrite results are reported as the mean concentration of nitrite (μM) ± SD of three independent experiments in triplicate. iNOS levels are normalized to β-actin ± SD. The blot is representative of four independent experiments. *n* = 4 corneas/group; * *p* < 0.05, ** *p* < 0.01, *** *p* < 0.001, **** *p* < 0.0001.

In the infected cornea, many different cell types function together in response to the invading pathogen. In vitro studies were carried out to complement the in vivo work regarding how Tβ4 may be influencing MΦ activation and function without the confounding factors of other inflammatory cells or the bacteria. Two time points (6 and 24 h) were assessed to evaluate the effect of Tβ4 on LPS-induced NO production and iNOS levels using RAW 264.7 cells (Figure 5). After 6 h of LPS stimulation, nitrite levels (A) were significantly elevated compared to media only. Tβ4 and ciprofloxacin groups remained comparable to controls, while the adjunctive Tβ4 treatment significantly decreased LPS-induced NO levels. At 24 h after LPS stimulation, NO production was significantly downregulated in the Tβ4 and adjunctive Tβ4 treatment groups, while no differences were observed after ciprofloxacin alone treatment. From 6 to 24 h, the results indicated that NO generation was increasing in MΦ exposed to LPS, but that Tβ4 also began to exert its function in both Tβ4 alone and adjunctive groups. The critical enzyme iNOS, responsible for NO generation from MΦ, was also detected at both time points (B). iNOS was expressed without differences among all groups at 6 h after treatment. However, at 24 h, there was a significant upregulation of iNOS following LPS stimulation compared to media only. Notably, adjunctive Tβ4 treatment significantly inhibited LPS-induced iNOS expression by MΦ. These results reveal that Tβ4 treatment—either alone or as an adjunct—effectively inhibits RNS generation by MΦ. 

### 2.6. Adjunctive Tβ4 Treatment Influences Markers of Efferocytosis to Improve Immune Response 

**Figure 6:**
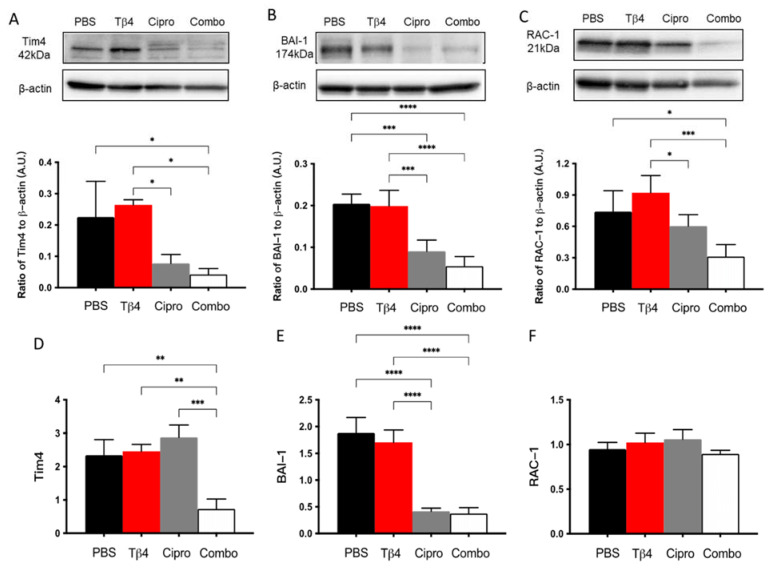
Efferocytosis activity was determined using select markers as detected at the protein level by Western blot (**A**–**C**) and mRNA level using real-time RT-PCR (**D**–**F**). Tim4 (**A**,**D**), BAI-1 (**B**,**E**), and RAC-1 (**C**,**F**) were assessed in corneas of PBS-, Tβ4-, cipro-, and combo-treated B6 mice at 3 days after infection. Western blot results are presented as a ratio normalized against β-actin ± SD. mRNA results are represented as a relative fold-change for the gene of interest compared to normal (uninfected) controls ± SD. *n* = 3 corneas/group; * *p* < 0.05, ** *p* < 0.01, *** *p* < 0.001, **** *p* < 0.0001.

MΦ engulfment of apoptotic PMN has been demonstrated as an essential event in the resolution of inflammation [30,31]. To further investigate the underlying mechanism(s) behind the improved disease response observed in the adjunctive Tβ4 treatment group, protein levels for select molecules known to be involved in MΦ efferocytosis were detected [30,31]: two cell surface receptors, Tim4 and BAI-1, and the signaling molecule RAC-1 (Figure 6). The results indicate that protein levels of Tim4, BAI-1, and RAC-1 were all significantly inhibited in the combination group, with no differences detected between Tβ4 alone and PBS control groups. It was also noted that ciprofloxacin only treatment significantly downregulated BAI-1 compared to the PBS group. mRNA levels were also determined to confirm whether these changes were also occurring at the transcriptional level. Tim4 (D) and BAI-1 (E) transcripts were significantly downregulated in the combination-treated corneas compared to PBS controls. No differences were observed between groups for RAC-1 (F) mRNA expression. Further, ciprofloxacin treatment was shown to significantly reduce mRNA levels of BAI-1 only, despite reduced protein levels for all three molecules.

### 2.7. Tβ4 Treatment Suppresses Markers of MΦ Efferocytosis Activity In Vitro

**Figure 7:**
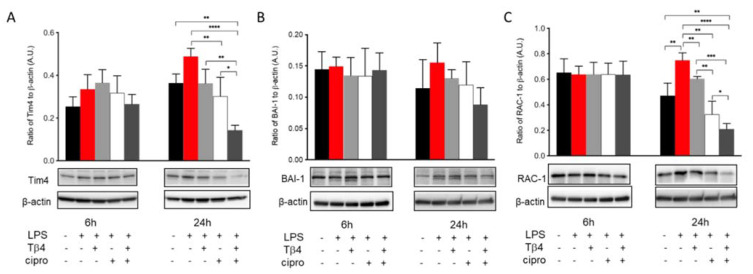
In vitro assessment of efferocytosis activity following LPS-induced stimulation of MΦ. Efferocytosis activity was measured from RAW 264.7 cells by Western blot at 6 and 24 h after LPS stimulation. Tim4 (**A**), BAI-1 (**B**), and Rac-1 (**C**) levels are presented as a ratio to β-actin ± SD. *n* ≥3 corneas/group; * *p* < 0.05, ** *p* < 0.01, *** *p* < 0.001, **** *p* < 0.0001.

The efferocytotic activity of MΦ was also analyzed in vitro to further confirm the regulatory influence of adjunctive Tβ4 treatment on MΦ cellular function in response to LPS stimulation. Levels of Tim4 (A), BAI-1 (B), and Rac-1 (C) in LPS-stimulated MΦ were detected after 6 and 24 h, as shown in Figure 7. The results revealed that Tim4 (A) expression was comparable among groups at 6 h. However, at 24 h, ciprofloxacin treatment significantly decreased LPS-induced Tim4 levels. More remarkably, adjunctive Tβ4 treatment significantly decreased LPS-induced Tim4 production when compared to all four groups. Similarly, BAI-1 (B) was uniformly expressed between all groups at 6 h after LPS stimulation. Despite trending upward after LPS stimulation and downward with the adjunctive treatment, no statistically significant differences were observed after 24 h. The signaling molecule, Rac-1 (C), was evenly expressed among all groups at 6 h. However, it was significantly increased after LPS stimulation at 24 h. LPS-induced levels of Rac1 were significantly reduced following Tβ4 alone and ciprofloxacin alone treatments, while the combination group resulted in a significant decrease in Rac1 when compared to all treatment groups. Together, these in vivo and in vitro data confirm that both Tβ4 and ciprofloxacin suppress efferocytotic functions of the MΦ.

## 3. Discussion

*Pseudomonas aeruginosa* is a common virulent, opportunistic pathogen associated with rapid progressing liquefactive necrosis of the cornea, often resulting in opacification and blindness [1]. However, host-associated factors largely determine the extent of the inflammatory response and subsequently, the disease outcome. In addition to effectively eliminating the bacteria, the host immune response should be modulated toward resolution to alleviate the adverse effects of sustained inflammation on corneal structure and function [32]. Developing an immunomodulatory agent that appropriately regulates infiltrating leukocytes, prevents corneal scar formation, and promotes corneal wound healing without side effects remains challenging. Sosne et al. have demonstrated that Tβ4 is an anti-inflammatory agent by suppressing nuclear translocation of the NF-κB pathway [13]. Additional studies have also demonstrated improved wound healing when administrating Tβ4 treatment in models of dry eye and alkali burn [12,17,33]. Our previous work expanded upon these results and illustrated that Tβ4 works synergistically with ciprofloxacin to enhance the therapeutic efficacy of antibiotics against corneal infection through modulation of inflammatory cells and specialized pro-resolving mediator pathways [28]. The current study further examined the regulatory effects of Tβ4, leading to enhanced host defense against *P. aeruginosa*-induced corneal infection in the susceptible B6 mouse model. 

While the exact mechanisms whereby Tβ4 exerts its inflammatory influence are only superficially understood, we provide evidence suggesting that Tβ4 carries out its profound effects, in large part, by affecting MΦ infiltration, activation, and function in the infected cornea, thus contributing to markedly improved disease outcome when combined with ciprofloxacin. MΦ are essential first-line defenders of the innate immune system [22], but need to be appropriately controlled. In individuals with overactive, chronically persistent MΦ influx and activation, the immune system is uncontrolled, leading to high levels of systemic inflammation and resultant organ damage. However, local depletion of MΦ using liposomal delivery of dichloromethylene diphosphonate has been shown to substantially inhibit the immune response, thus exacerbating disease in a mouse model of keratomycosis [34]. Ideally, when the immune system is activated, MΦ fight against the infection in an organized and controlled manner [22] with a prompt shift toward resolution. The results from the current study indicate that adjunctive Tβ4 treatment influences MΦ in such a way: suppression of excessive cellular infiltration, inhibition of pro-inflammatory activation, while enhancing anti-inflammatory/pro-resolving markers, reduction of inflammation-induced expression of nitric oxide, and regulating efferocytosis. 

While increased cellular infiltration and excessive generation of pro-inflammatory mediators are more evident components leading to worsened disease, RNS produced by MΦ also play an important role in the pathogenesis of microbial keratitis. In fact, controlled production of RNS is essential in inhibiting inflammation, promoting resolution, and restoring tissue homeostasis [28,29]. A report from Hazlett et al. has shown that BALB/c mice treated with aminoguanidine sulfate (AG), an inhibitor of iNOS, led to decreased nitrite levels. This was accompanied by increased bacterial burden and elevated inflammatory markers derived from MΦ, resulting in substantial corneal devastation, suggesting that iNOS-derived NO is required for bacterial killing/stasis [29]. However, excess production of NO has been implicated in the pathogenesis of inflammation [35]. Three isoforms of nitric oxide synthase (NOS) have been reported and include neuronal (n) NOS, inducible (i) NOS, and endothelial (e) NOS. However, MΦ are one of the best-characterized sources of iNOS induced by microbial infections [29]. iNOS activity has been shown to increase in a model of endotoxin-induced uveitis (EIU), and when NOS activity was inhibited, the inflammatory response was blunted. Moreover, it has been shown that NO inhibition alleviates the clinical signs of EIU [36]. Another study of experimental keratitis in rabbits revealed that NO plays an essential role in disease pathogenesis and that NO inhibition resulted in enhanced host defense [37]. Results of the current study showed that adjunctive Tβ4 treatment significantly decreased NO generation and iNOS activity in corneas following *P. aeruginosa*-induced keratitis. These findings correlate with our previous work showing that Tβ4 reduces corneal iNOS mRNA expression at both 3 and 5 days p.i. [28]. It is worth noting that while NO levels were not as downregulated in corneal lysates as expected given the markedly reduced enzymatic levels of iNOS, enzymatic activity of eNOS and nNOS could be contributing to NO generation detected within the whole cornea, as well. In partial response to this possibility and to further complement the current work, in vitro studies were carried out to remove the influence of other inflammatory cells known to produce ROS/RNS. Moreover, given that Tβ4 does not appear to be antimicrobial in nature [28], stimulating with LPS allowed for better understanding of how Tβ4 modulates inflammation without the confounding factor of infectious bacteria. As a result, it is further demonstrated that LPS-induced NO and iNOS levels were reduced with adjunctive Tβ4 treatment as well as Tβ4 alone. These findings are not only important in that RNS drives inflammation, but endogenous RNS together with ROS act as signaling molecules to modulate phagocytosis and apoptosis [38]. As a result, Tβ4 may also influence those signaling molecules that regulate PMN function. 

There is abundant evidence that apoptotic cells can suppress inflammation and that failure to clear apoptotic cells exacerbates the response, indicating that efferocytosis plays a crucial role in modulating the inflammatory response of MΦ to promote resolution [23,30,31,39]. Uptake of apoptotic bronchial epithelial cells and neutrophils is significantly reduced in bronchoalveolar lavage MΦ from patients with COPD compared to healthy controls [40,41,42]. Another study revealed that efferocytosis plays a dual role in lung infections. While efficient uptake of apoptotic cells is required to resolve the inflammatory response, the anti-inflammatory programs that are activated upon prolonged exposure to apoptotic cells might increase susceptibility to secondary infections and exacerbate chronic inflammatory lung diseases [43]. Nevertheless, the function of efferocytosis in keratitis is not as well-characterized. In this regard, neither PtdSer recognition receptors (e.g., BAI-1, Tim4) nor signaling molecules (e.g., RAC-1) have been studied in the keratitis model. The current work begins to understand how efferocytosis is regulated in the eye. The prolonged upregulation of Tim4, BAI-1, and RAC-1 in PBS and Tβ4-treated corneas revealed that MΦ were, most likely, actively working to clear the large population of apoptotic PMN and other cellular debris as a result of the ongoing, robust inflammatory response. On the other hand, the diminished efferocytotic activity observed in the adjunctive Tβ4 treatment group was consistent with the very mild disease response and correlates with low levels of apoptotic PMN as well. 

The differences observed between in vitro and in vivo studies emphasize our previous findings that Tβ4 does not appear to function as an antibacterial and, as a result, fails to exert its regulatory effects to full capacity in the presence of bacteria. By omitting the bacteria from the in vitro studies, Tβ4 alone was demonstrated to regulate the MΦ response to LPS-induced inflammation, as shown by reduced RNS generation and efferocytosis marker expression. In fact, our findings show that Tβ4 acts as a transcription modulator, as indicated by changes at both the mRNA and protein levels [28]. Our previous work also revealed a synergistic effect between Tβ4 and ciprofloxacin on lipoxygenase expression [27], key enzymes involved in the resolution circuit of inflammation. It has also been previously reported that Tβ4 acts on cell surface ATP synthase to increase extracellular ATP levels, which in turn activate P2X7R to upregulate Ca^2+^ influx and trigger phosphorylation of ERK1/2, resulting in proliferation and migration of human corneal epithelial cells [44]. In addition, P2X7R is a known activator of the inflammasome and widely expressed on MΦ to modulate the immune response [45]. P2X7R stimulates MΦ to release potent anti-inflammatory proteins, such as annexin A1 [46], suggesting a potential role for this receptor during resolution of inflammation following Tβ4 treatment. The current work further highlights this synergistic relationship on RNS generation and markers of efferocytosis. These collective results support that the improved disease response observed in corneas and cells treated with Tβ4 + ciprofloxacin stems from their synergistic regulation of MΦ infiltration, activation, and function. Likewise, the findings regarding ciprofloxacin alone were notable as well. Treatment with ciprofloxacin only significantly decreased the expression of infiltrated leukocytes and MΦ, resulting from antimicrobial effects leading to fewer bacteria present within the infected cornea. However, the in vitro results reveal ciprofloxacin-induced effects on the host response extending beyond the expected bacterial killing, as exhibited in vivo. Enhanced levels of pro-inflammatory markers and reduced RNS generation by MΦ reflect unexpected, off-target effects of ciprofloxacin. In fact, ciprofloxacin had to be reduced from 0.3% to 0.03% for our in vitro studies due to toxicity issues indicated by excessive cell death in the presence of the antibiotic at the former concentration. These findings together establish a basis for further studies on the functional activities of ciprofloxacin regarding the host response given its widespread use in the clinical setting [27].

Collectively, these findings suggest that Tβ4 treatment has a minimal effect when used alone in vivo. However, adjunctive Tβ4 with ciprofloxacin substantially enhances and regulates the host defense against inflammation, resulting in markedly improved disease outcome. As Tβ4 has no reported adverse effects, this work strongly supports its use as an adjunct treatment for bacterial keratitis to alleviate the drawbacks of the current standard of care. 

## 4. Materials and Methods 

### 4.1. Experimental Animal Protocol

Eight-week-old female C57BL/6 (B6) mice were purchased from The Jackson Laboratory (Bar Harbor, ME, USA). Following wounding of the cornea using established methods, a 5 μL aliquot of bacterial suspension containing 10^6^ CFU/mL of the cytotoxic *Pseudomonas aeruginosa* strain ATCC 19,660 (Manassas, VA, USA) was immediately applied to the corneal surface, as previously described [47]. Mice were then randomized into four different treatment groups: PBS as controls, Tβ4 (0.1%) (Regenerx Biopharmaceuticals Inc., Rockville, MD, USA), ciprofloxacin (0.3%), or Tβ4 + ciprofloxacin, which were administered topically (5 μL) 3× per day beginning 24 h after infection until animals were euthanized at 3 days p.i. All animals were treated in a manner authorized by Wayne State University Institutional Animal Care and Use Committee (protocol 19-10-1312) and conformed to the Association for Research in Vision and Ophthalmology’s statement on the Use of Animals in Ophthalmic and Vision Research (8th edition).

### 4.2. Clinical Scoring

After infection, the corneas were observed daily in a blinded fashion and were graded using an established grading scale [48]. A clinical score was recorded at 3 days post-infection (p.i.) for each mouse (*n* = 11 per group per treatment) to express disease severity and presented as mean clinical score ± SEM for each experimental group: 0 = clear or slight opacity, partially or fully covering the pupil; +1 = slight opacity, fully covering the anterior segment; +2 = dense opacity, partially or fully covering the pupil; +3 = dense opacity, covering the entire anterior segment; +4 = corneal perforation [48]. 

### 4.3. Cell Culture and Treatment

Murine monocyte/MΦ-like RAW 264.7 (ATCC; TIB-71) cells, initially derived from BALB/c mice, were cultured in DMEM containing 10% heat-inactivated FBS (Invitrogen Life Technologies, Carlsbad, CA, USA) and penicillin (100 U/mL) at 37 °C and 5% CO_2_. Before treatment, cells were seeded in 6-well plates at a density of 0.2 × 10^6^ cells/well (total volume of 2 mL) and divided into four different groups: media only/no treatment (positive control), Tβ4 (0.1% final concentration), ciprofloxacin (0.03% final concentration), and combination (0.1% Tβ4 and 0.03% ciprofloxacin final concentrations). Once cells became confluent, all four groups were stimulated with lipopolysaccharide (PA, serotype 10-derived LPS, Sigma-Aldrich, St. Louis, MO, USA) for up to 24 h. Media only/no treatment without LPS stimulation served as the negative control. At both 6 and 24 h, cell supernatants and lysates from each group were collected for analysis.

### 4.4. Flow Cytometric Analyses

Individual corneas from each treatment group were harvested at 3 days p.i. in sterile tubes containing 250 µL of RPMI 1640 without serum. Single-cell suspensions were obtained as previously described [49]. Cell pellets were washed and resuspended using FACS buffer (1% BSA in PBS). After performing cell counts and viability using trypan blue, cells were incubated with antibodies (listed below) along with a fixable viability cell stain and staining buffer at 4 °C for 30 min. Cells were washed twice with FACS buffer, and the pellets were re-suspended in 1 mL of cold FACS buffer. Samples were immediately acquired using a flow cytometer (LSRFortessa; Beckton Dickinson, San Jose, CA, USA). Data were analyzed using FlowJo software. The following antibodies were used for cell surface staining: AF405-conjugated rat anti-mouse CD13 (R3-63, 1:200) (Novus Biologicals, Centennial, CO, USA), PerCP-conjugated rat anti-mouse CD45 (30-F11, 1:200), PE-conjugated rat anti-mouse CD63 (NVG-2, 1:200), AF647-conjugated rat anti-mouse CD177 (Y127, 1:200) (BD Biosciences, San Jose, CA, USA), APC-conjugated hamster anti-mouse CD80 (16-10A1, 1:200), APC-conjugated rat anti-mouse CD86 (GL-1, 1:200), FITC-conjugated rat anti-mouse CD195 (HEK/1/85a, 1:200), PE-Cy7-conjugated rat anti-mouse CD206 (MR6F3, 1:200), LIVE/DEAD^TM^ Fixable Aqua dead cell stain (L34965) (ThermoFisher Scientific, Rockford, IL, USA), BV785-conjugated rat anti-mouse CD192 (SA203G11, 1:200), APC-R700-conjugated rat anti-mouse F4/80 (BM8, 1:200), and APC-Fire 750-conjugated rat anti-mouse Ly-6G (1A8, 1:200) (BioLegend, San Diego, CA, USA).

### 4.5. Griess Reaction

NO production was measured (as previously described [8]) as its stable end product, nitrite, using the Griess reagent (1% sulfanilamide/0.1% naphthyl ethylene diamine dihydrochloride 12.5% H_3_PO_4_) in the corneas of PBS-, Tβ4-, ciprofloxacin-, and Tβ4 + ciprofloxacin-treated B6 mice. In brief, corneas were homogenized in 500 µL of degassed PBS and micro-centrifuged at 3500 rpm for 5 min. Next, 100 µL of supernatant was added to an equal volume of Griess reagent in triplicate in a 96-well microtiter plate and incubated at room temperature for 15 min. Absorbance at 570 nm was measured and nitrite concentrations were estimated against a sodium nitrite standard curve. Data were reported as the mean μM/cornea concentration of nitrite ± SD.

### 4.6. Western Blot

Infected corneas were excised at 3 days p.i. from all treatment groups and homogenized in 200 µL of RIPA buffer (Cell Signaling Technology, Danvers, MA, USA). For in vitro studies, RAW 264.7 cells were collected at both 6 and 24 h after treatment and lysed in M-PER buffer (Thermo Fisher Scientific, Waltham, MA, USA). A protease inhibitor cocktail (Thermo Fisher Scientific, Waltham, MA, USA) was added to both the corneal samples and cell lysates. Then, the samples were sonicated to achieve complete lysis and centrifuged at 12,000 RPM for 20 min. Supernatants were collected and normalized for equal amounts of protein as determined by BCA methods, then separated onto 4–20% tris-glycine gels (Invitrogen, Carlsbad, CA, USA) and transferred to PVDF membranes. After blocking the membranes in 5% non-fat milk dissolved in TBST (10 mmol/L Tris-HCl buffer, pH 8.0, 150 mmol/L NaCl, and 0.1% Tween 20) at room temperature for 60 min, membranes were incubated overnight at 4 °C with antigen-specific primary antibodies. The primary antibodies were used as follows: anti-iNOS (1:1000; Abcam, Cambridge, UK), anti-Tim4 (1:500; Abcam, Cambridge, UK), anti-BAI1 (1:800; Invitrogen, Carlsbad, CA, USA), anti-RAC-1 (1:1000; Invitrogen, Carlsbad, CA, USA), and anti-β-actin (1:1000; Santa Cruz Biotechnology, Dallas, TX, USA). They were then followed by incubation with species-specific horseradish peroxidase-conjugated secondary antibodies for 1 h at room temperature. Proteins were visualized by incubation with a chemiluminescence substrate kit (Thermo Fisher Scientific, Waltham, MA, USA). Western blot images were collected (Bio-Rad Molecular Imager, ChemiDoc XRS+), and target protein expression was quantified using Image Studio Lite software version 5.2 (LI-COR Biosciences, Lincoln, NE, USA), normalizing to β-actin. All the antibodies were repeated at least three times, and one representative blot was shown for each molecule.

### 4.7. Real-Time RT-PCR

Total RNA was isolated from individual whole corneas for gene expression analysis using RNA-STAT 60 (Tel-Test, Friendswood, TX, USA), according to the manufacturer’s recommendations, and quantified by spectrophotometric determination (260 nm). cDNA templates were constructed by reverse transcribing 100 ng of total RNA, then amplified using SYBR^®^ Green Master Mix (Thermo Fisher Scientific, Waltham, MA, USA), per the manufacturer’s instruction, with the reaction mixture previously described [49]. All primers were generated using Primer3 PCR v. 4.1.0 primer design software, and primer sequences are shown in Table 1. Semi-quantitative real-time RT-PCR was carried out using the CFX Connect Real-Time RT-PCR Detection System (BioRad, Hercules, CA, USA). Changes in mRNA expression were calculated using the relative standard curve method comparing the amount of target normalized to an endogenous reference, β-actin [50]. Results are reported as the mean fold change ± SD normalized to β-actin and relative to the expression of uninfected (normal) corneas. 

### 4.8. Statistical Analysis

Sample sizes were determined statistically before experimentation based on previous work. All experiments were carried out from a minimum of three independent experiments, and representative data from a typical experiment are shown. Data are presented as mean ± SD unless otherwise noted. All data were analyzed by the one-way ANOVA followed by Bonferroni’s multiple comparison test (GraphPad Prism, San Diego, CA, USA). Data were considered significant at *p* < 0.05.

## Figures and Tables

**Table 1:** Nucleotide sequence of the specific primers used for PCR amplification.

Gene	Nucleotide Sequence	Primer
β-actin	5′-ACTGGGAGACATGGAGAAG-3′	F
	5′-GTCTCCGGAGTCCATCACAA-3′	R
Tim4	5′-GGGTGTACTGCTGCCGTATA-3′	F
	5′-TCACTGCTGTACTGAAGGCA-3′	R
BAI-1	5′-CACTTGCTTACCCACCCTTG-3′	F
	5′-AGCTCATCCCCAAACTCCTC-3′	R
RAC-1	5′-GCTCATCAGTTACACGACCA-3′	F
	5′-GTAGGAGAGGGGACGCAATC-3′	R
